# Biological reconstruction of bone defect after resection of malignant bone tumor by allograft: a single-center retrospective cohort study

**DOI:** 10.1186/s12957-023-03121-7

**Published:** 2023-07-31

**Authors:** Qing Liu, Feng Long, Can Zhang, Yupeng Liu, Hongbo He, Wei Luo

**Affiliations:** 1 Department of Orthopaedics, Xiangya Hospital, Central South University, 87Th Xiangya Road, Changsha, 410008 Hunan China; 2 National Clinical Research Center for Geriatric Disorders, Xiangya Hospital, Changsha, Hunan 410008 People’s Republic of China

**Keywords:** Allograft, Bone defect repair, Malignant bone tumor, Internal fixation, Bone healing, Long-bone metaphysis

## Abstract

**Background:**

Allograft reconstruction following the resection of malignant bone tumors is associated with high rates of complications and failures. This study aimed to evaluate the efficacy and current problems of allograft reconstruction techniques to optimize treatment strategies at our center.

**Materials and methods:**

Thirty-eight cases (16 men and 22 women), who were diagnosed with malignant bone tumors and had undergone allograft reconstruction, were recruited. Allograft was fixed by intramedullary nail, single steel plate, double plate, and intramedullary nail combined plate in 2, 4, 17, and 15 cases, respectively. Allograft union, local recurrence, and complications were assessed with clinical and radiological tests. Tumor grade was assessed using the Enneking staging of malignant bone tumors. Functional prognosis was evaluated by the Musculoskeletal Tumor Society (MSTS) scoring system.

**Results:**

Intercalary and osteoarticular reconstructions were performed in 32 and 6 cases, respectively. Six patients underwent reoperation related to allograft complications, four patients had local recurrence, and three patients with allograft fracture underwent allograft removal. A total of eight host–donor junctions showed nonunion, including seven cases (18.4%) in diaphysis and one case (3.1%) in metaphysis (*p* < 0.01). Host rejection and secondary osteoarthritis occurred in nine and two cases, respectively. No deep infection and internal fixation device fracture occurred. The overall allograft survival rate was 81.6%. Postoperative MSTS score of patients with allograft survival was 26.8 ± 2.9, indicating a significant improvement as compared to their preoperative function.

**Conclusions:**

Allograft represents an excellent choice for intercalary bone defects after malignant bone tumor resection. Robust internal fixation protection across the whole length of the allograft is an important prerequisite for the survival of the allograft, while multidimensional osteotomy, intramedullary cement reinforcement, and pedicled muscle flap transfer can effectively improve the survival rate and healing rate of the allograft.

**Supplementary Information:**

The online version contains supplementary material available at 10.1186/s12957-023-03121-7.

## Introduction

Along with the widespread application of neoadjuvant chemotherapy, radiotherapy, and targeted drug therapy for the clinical treatment of malignant bone tumors, advances in imaging technology have enabled precise boundary determination and accurate resection of these tumors, resulting in more frequent limb-salvage treatment and maximal improvement of life expectancy and restoration of limb function [1–3]. However, in the context of an enthusiastic paradigm shift toward limb-salvage surgery, selecting the most appropriate reconstruction method after tumor resection remains challenging and controversial [4–9].

The reconstruction options for large bone defects after bone tumor resection primarily include mechanical [10, 11] and biological reconstruction [12–14]. Mechanical reconstruction refers to the use of artificial metal prostheses to repair bone defects and restore bone structure integrity. Despite its favorable short-term prognosis, its long-term outcome is not satisfactory owing to elastic modulus mismatch, prosthesis loosening, and limited service life of the prosthesis [10, 11]. Therefore, these problems should be urgently addressed in mechanical reconstruction. Biological reconstruction involves the use of various biomaterials to restore the structural integrity and biological activity of bone defects. This approach is more consistent with the requirements of human biomechanics. Compared to mechanical reconstruction, biological reconstruction has more short-term complications and a longer recovery period [15–17]. However, once biological reconstruction is successful, lifelong benefits are expected. Additionally, biological reconstruction failure caused by factors other than tumor recurrence can still be repaired by mechanical reconstruction without loss of host bone mass, which is a unique advantage of this reconstruction method. With the rapid development of comprehensive oncology treatments, the life expectancy of cancer patients has significantly improved. Therefore, in-depth research on the repair of large bone defects after bone tumor resection is beneficial.

In different patients, the optimal selection of different reconstruction methods considering their advantages and disadvantages is at the discretion of clinicians. Large-segment allografts have been used in clinical practice for decades as a biological reconstruction method for bone defect repair. However, the clinical efficacy of this method varies among treatment centers, and there is no consensus or guidance on its specific scope of application and surgical techniques [17–19]. This study aimed to retrospectively analyze data from our bone tumor center on the use of large-segment allografts to repair bone defects after resection of malignant bone tumors and comprehensively evaluate the scope of its application and its efficacy in oncology prognosis, functional prognosis, and complications. Additionally, this study aimed to provide supporting data and a theoretical basis for the rationalization and standardization of allograft application for the repair of bone defects.

## Materials and methods

### Patients

The study was conducted in accordance with the Declaration of Helsinki and was approved by the clinical medical research ethics committee of Xiangya Hospital of Central South University (201,205,032). All the patients participating in the study or their legal guardians signed an informed consent form.

We retrospectively analyzed the clinical data of patients treated with massive allografts between January 2012 and January 2022. The inclusion criteria were as follows: the presence of histopathologically confirmed primary malignant bone tumor, bone defect repair with a massive allograft, and complete clinical data with long-term follow-up of > 24 months. This cohort consisted of 38 patients (16 men and 22 women) with an average age of 23.1 ± 13.7 years. The lesions were in the femur in 20 cases, tibia in 13 cases, humerus in 3 cases, and radius and ulna in 1 case each. Pathological diagnoses included osteosarcoma (*n* = 22), chondrosarcoma (*n* = 3), Ewing’s sarcoma (*n* = 6), undifferentiated pleomorphic sarcoma (*n* = 1), ameloblastoma (*n* = 2), and fibrosarcoma (*n* = 4). All patients were preoperatively examined using radiography, computed tomography (CT), magnetic resonance imaging (MRI), and radionuclide bone imaging to evaluate their general condition and lesion boundary (Supplemental table). Tumor grade was assessed by Enneking staging of malignant bone tumors [20], and limb functional evaluation was performed using the International Society of Limb Salvage and the Musculoskeletal Tumor Society (MSTS) score [21]. All data were obtained from the medical records, radiographs, and outpatient follow-up evaluations.

### Therapeutic procedure

According to the results of the pathological diagnosis, neoadjuvant chemotherapy was administered to all patients, except for those with chondrosarcoma and ameloblastoma. The actual lesion boundary was determined according to T1WI-enhanced MRI images before chemotherapy. The patients underwent radical resection as follows: the surgery began with resection of the initial biopsy channel, removal of a suitable soft tissue cuff, and osteotomy at least 3 cm from the proximal and distal ends of the tumor bone. The lesion near the joint was removed via intra-articular resection. To achieve the closest anatomical matching, we selected allografts based on a comparison of X-ray and CT scans of patients and donors. The gradient rewarming method was performed to rewarm the allograft, which was soaked in ice-cold saline for 10 min, normal temperature saline for 10 min, and hydrogen peroxide for 15 min. The bone marrow tissue and cancellous bone in the allograft cavity were thoroughly cleaned with a curette and rinsed repeatedly, and allografts of the same length were cut to repair the bone defects. V-shaped or stepped osteotomy was performed for all allograft-host junctions. The allograft fixation methods included a single steel plate (Stryker, Michigan, USA), double steel plate, intramedullary nail (Stryker, Michigan, USA), and intramedullary nail composite short steel plate. The average length of allograft was 16.5 cm, with 32 cases of intercalary allograft (including 7 cases of arthrodesis) and 6 cases of osteoarticular allograft. Almost all the patients required pedicled muscle flap transfer for allograft and internal fixation coverage. Excellent soft-tissue coverage is a critical step in reducing infection and promoting allograft activation. At least two wound drainage tubes were placed, and first-generation cephalosporins were administered to prevent infection until the drainage tubes were removed. Patients with osteoarticular allografts were assisted with external splints or braces for 4–6 weeks postoperatively.

### Postoperative management

Physiotherapists instructed the patients to use braces, walk with crutches, and contract the affected muscles. Isometric contraction exercise was started 1 week after operation, and passive functional exercise was started 2 weeks after operation, under the guidance of physiotherapists. The patients were informed about walking with crutches and non-weight-bearing standing for the first 6 weeks. Partial and full weight bearing gradually started after the imaging examination confirmed the healing tendency of the fracture. Patients were followed-up at 6 and 12 weeks postoperatively and then every 3 months for the first 2 years, every 6 months for the next 3 years, and annually thereafter. Follow-up mainly evaluated oncological prognosis, bone healing, and functional prognosis and recorded the occurrence of various complications. Removal or replacement of massive allografts resulted in treatment failure. Bone healing was considered when the osteotomy line was no longer visible or the junction was bridged with the periosteal bone on two orthogonal radiographic views.

### Statistical analysis

Data analysis was performed using SPSS version 20 (SPSS Inc., Chicago, IL, USA). The measurements are expressed as mean ± standard deviation. Follow-up data were analyzed using paired or unpaired *t*-tests, and categorical variables were compared using Fisher’s exact test. Survival estimates for the allografts were obtained using the Kaplan–Meier method. Statistical significance was set at *p* < 0.05.

## Results

### Oncologic prognosis and allograft survival

**Fig. 1:**
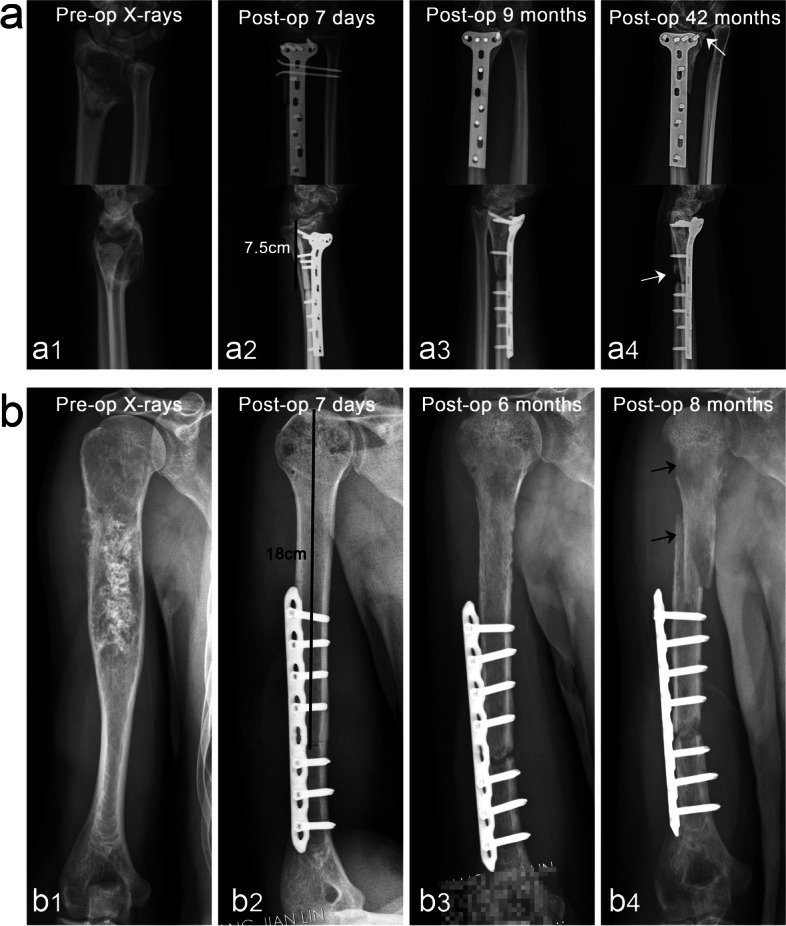
Complications of bone and joint allograft (cases 23 and 38). In the patients with stage IIB fibrosarcoma of distal radius, stage IIB chondrosarcoma of proximal humerus was treated with allograft bone joint transplantation to repair bone defects. a1 Preoperative anteroposterior and lateral X-rays. a2–a4 Continuous follow-up of X-ray after surgery, the proximal part of the allograft gradually developed bone resorption leading to bone nonunion, and the distal carpal surface developed secondary osteoarthritis (white arrows indicate sites of nonunion and osteoarthritis, respectively). b1 Preoperative anteroposterior X-rays. b2–b4 The length of the allograft was 18 cm. Follow-up revealed that the allograft was gradually absorbed, with nonunion of the host–donor junction, loosening of the internal fixation, and eventually the allograft fracture occurred (black arrows indicate sites of bone resorption and fracture location, respectively)

Until the last follow-up, four cases (10.5%) underwent amputation due to local recurrence at an average of 35.7 months after surgery, and all died of lung metastasis, whereas another five cases survived with lung metastasis. The overall survival rate was 89.5%. We identified 10 patients who required secondary surgery, including 4 with amputation due to tumor recurrence, 1 with revision due to secondary osteoarthritis, 3 with allograft removal due to allograft fracture (Fig. 1), and 2 with bone grafting due to host–donor junction nonunion. Indications for reoperation included nonunion or delayed union of the host–donor junctions, allograft fracture, and secondary osteoarthritis (Table 1). The overall allograft survival rate was 81.6% (Supplemental figure).

**Table 1:** Demographic and clinical information of patients

General information	Minimum	Maximum	Mean	SD
**Age**	13	66	23.1	13.7
**Allograft length (cm)**	12	73	16.5	3.2
**Duration of follow-up (month)**	7.5	25	57	23.9
**General information**			**Number**	**Percentage**
***Gender***	M		16	42.1%
	F		22	57.9%
***Anatomical location***	Femur		20	52.6%
	Tibia		13	34.2%
	Humerus		3	7.9%
	Radius		1	2.6%
	Ulna		1	2.6%
***Complication***	Relapse		4	10.5%
	Metastasis		9	23.7%
	Nonunion		8	21.1%
	Rejection		9	23.7%
	Fracture		3	7.9%
	Osteoarthritis		2	5.3%
***Diagnosis***	Osteosarcoma		22	57.9%
	Chondrosarcoma		3	7.9%
	Ewing’s sarcoma		6	15.8%
	UDPS		1	2.6%
	Ameloblastoma		2	5.3%
	Fibrosarcoma		4	10.5%
***Enneking stage***	IIA		7	18.4%
	IIB		31	81.6%
***Reconstruction technique***	Intercalary		32	84.2%
	Osteoarticular		6	15.8%
***Interface matching***	Rough		18	47.4%
	Precise		20	52.6%
***Method of fixation***	IMN		2	5.3%
	INCP		15	39.5%
	DP		17	44.7%
	SSP		4	10.5%

*IMN* intramedullary nail, *SSP* single steel plate, *DP* double plate, *INCP* intramedullary nail combined plate, *UDPS* undifferentiated pleomorphic sarcoma

### Allograft union

**Fig. 2:**
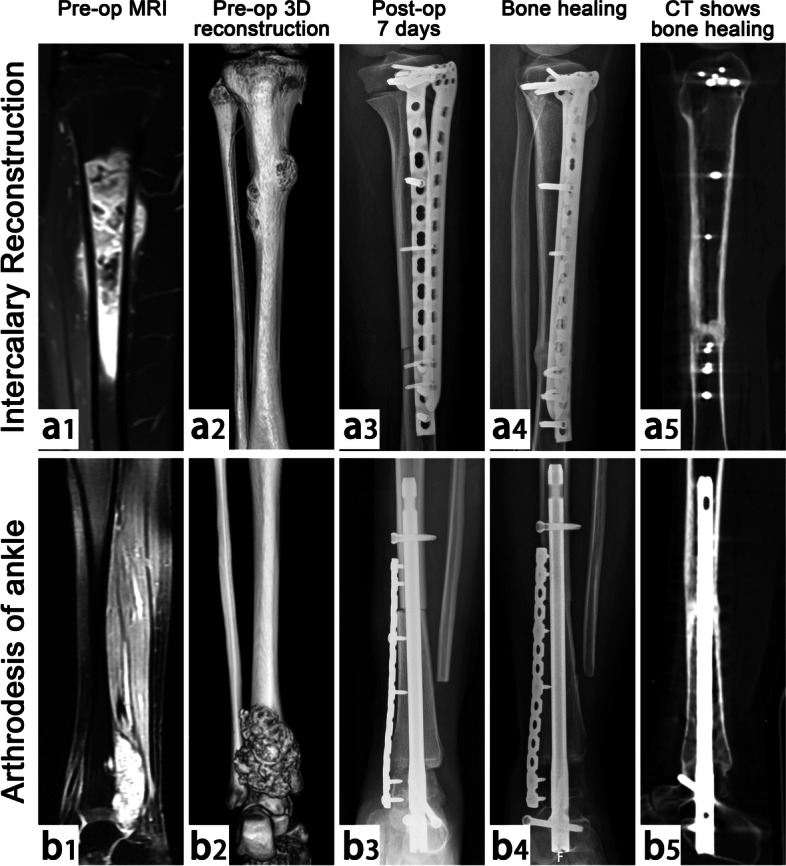
Limb salvage in 2 patients with stage IIB osteosarcoma of proximal tibia and distal tibia (case 2 and case 5). a1 and b1 Preoperative MRI examination and a2 and b2 CT 3D reconstruction graphics. a3 and b3 At postoperative 7 days, X-ray showed that the allograft matched well with the host and was firmly fixed. a4 and b4 At postoperative 16 months, X-ray showed that both junctions of the host–donor had healed, and no obvious loosening of internal fixation was found. a5 and b5 CT scan confirmed both junctions had healed completely

**Fig. 3:**
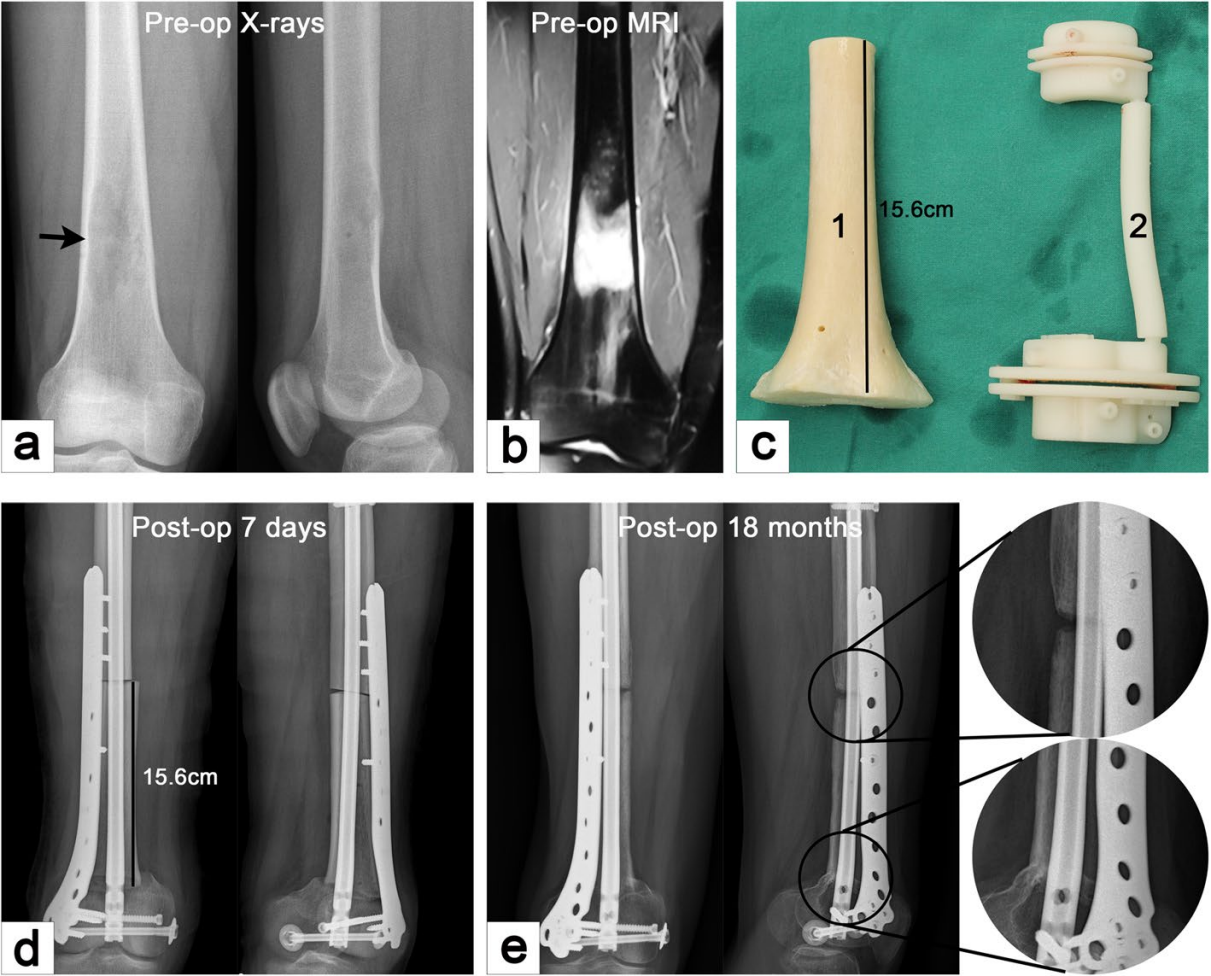
Case 21. A case of stage IIA osteosarcoma in distal femur. **a** and **b** Anteroposterior and lateral X-ray and MRI images. **c** Allograft for repairing bone defect and 3D-printed osteotomy guide plate. **d** and **e** Anteroposterial and lateral X-ray were performed at 7 days and 18 months postoperatively, respectively. The results showed that the host–donor junction of the metaphysis had achieved bone healing, but the junction at the diaphysis had nonunion, and there was no sign of loosening of internal fixation

In this cohort of 38 cases, there were 70 host–donor junctions, including 32 junctions at the metaphysis and 38 junctions at the diaphysis. The host–donor junction was completely healed in 30 cases (Fig. 2), with an average union time of 13 months, including 16 cases with three-dimensional (3D) printing-guided osteotomy. Follow-up showed that patients with 3D printing-assisted multidimensional osteotomy had a significantly improved bone healing time as compared to those with ordinary transverse osteotomy. Additionally, in four patients with tumor recurrence who required amputation, dissection of the gross specimen revealed significantly activated allograft surface with abundant neovascularization. Imaging examination showed that the host–donor junctions had healed. Hematoxylin and eosin staining showed that complete bone connection was formed with several new Haversian systems at the host–donor interface [22]. Nonunion or delayed union occurred in eight host–donor junctions (Fig. 3), including seven cases (18.4%) in diaphysis and one case (3.1%) in metaphysis (*p* < 0.01), indicating a significant difference in the healing capacity between the diaphysis and metaphysis. Two patients underwent bone grafting or screw compression reoperation at the host–donor junction to achieve bone union; three patients did not receive any interventions because the host–donor nonunion did not affect limb function (Video 1). Since all cases in this study were treated with postoperative chemotherapy or radiotherapy, we could not analyze the specific impacts of these treatments on bone healing.

### Functional outcome

The MSTS score before the reoperation was used as the final functional score. A total of 33 cases (86.8%) had good or excellent outcomes, and five (13.2%) had fair outcomes according to the MSTS score system. MSTS scores were given to patients whose allografts survived, with an average score of 27.6, indicating a significant functional improvement compared to the preoperative state (Table 2). Functional activity of the affected side was restored to > 90% of that of the healthy side (Video 2). All patients were able to resume their normal life. Most patients were able to return to their original jobs, with only three patients changing jobs due to limb function. Six patients presented unequal limb lengths (two in the upper limbs and four in the lower limbs), with an average limb length of 2.5 cm. Four patients with unequal lower limb lengths had normal force lines and increased insoles to assist in balancing the length of the lower limbs without any correction or extension of surgical treatment.

**Table 2:** Comparative statistical analysis of various factors

Comparative analysis	Potential factors	*N* (total)		*p*-value
***Nonunion***	Diaphysis	7 (38)		< 0.001
	Metaphysis	1 (32)		
	Intercalary	4 (32)		< 0.01
	Osteoarticular	4 (6)		
	Rough	7 (18)		< 0.001
	Precise	1 (20)		
***Fracture***	Intercalary	0 (32)		< 0.001
	Osteoarticular	3 (6)		
		**Mean**	**SD**	***p*-value**
***MSTS score***	Preoperative	19.2	3.5	< 0.001
	Postoperative	26.8	2.9	

*MSTS* Musculoskeletal Tumor Society scoring system

### Postoperative complications

Excluding tumor recurrence, 17 patients had postoperative complications, including early host rejection (*n* = 9), allograft fracture (*n* = 3), nonunion (*n* = 8), and secondary osteoarthritis (*n* = 2). When suspected rejection occurs, hormone therapy is usually administered after ruling out infection (dexamethasone, 5 mg/bid for adults and 5 mg/qd for adolescents for 3–5 days). Failures included four cases of amputation due to tumor recurrence and three cases of allograft fracture. Patients with allograft fractures eventually underwent autologous fibula transplantation with free fibula or artificial hinge knee arthroplasty. Moreover, one patient with secondary osteoarthritis of the wrist showed allograft absorption (Fig. 1). However, wrist function was still satisfactory (Video 3). The other patient with secondary osteoarthritis could not tolerate knee joint pain and underwent hinged knee replacement. Fortunately, there was no deep infection in any case, which may be a benefit of the excellent soft tissue coverage of the allograft through pedicled muscle flap transfer, adequate wound drainage, and adequate antibiotic treatment.

## Discussion

In the context of an enthusiastic paradigm shift toward limb-salvage surgery, selecting the most appropriate reconstruction method after tumor resection remains challenging and controversial. There are various surgical reconstruction options, including artificial prosthesis replacement [7], autogenous bone transplantation [5], allograft transplantation [23, 24], the Masquelet technique [8], bone lengthening [25], autogenous devitalized bone reimplantation [26], and autogenous fibula composite allogeneic bone transplantation [27]. Mechanical reconstruction with an artificial prosthesis is often suitable for older patients or patients with metastatic tumors because of its excellent short-term efficacy. It seems more reasonable for young patients with malignant bone tumors to undergo biological reconstruction. Allografts, as substitute materials, have high mechanical stability, strong bone conduction, and excellent osteoinductive ability. With the help of internal fixation, allograft can be used for a long time after healing with host bone, and its surface can be activated to realize the growth of blood vessels and be used for a lifetime. Therefore, an in-depth understanding of the scope of adaptation, surgical manipulation skills, and specific therapeutic effects of allograft repair of bone defects will contribute to the rationalization and standardization of allograft use to serve more patients with massive bone defects.

This study retrospectively analyzed data from our bone tumor center on the use of massive allografts to repair bone defects after tumor resection. The results showed that the overall allograft survival rate was 81.6%, which was similar to or even better than that reported in several previous studies [28–30]. The survival time of patients is the ultimate goal of limb-salvage treatment, and allograft repair of bone defects is more meaningful if the patient’s life span can be further extended. Consequently, in the selection of patients for limb salvage, we may subjectively choose patients who are more sensitive to neoadjuvant chemotherapy and have resectable surgical boundaries detected by imaging examination. Conversely, if patients had no response to neoadjuvant chemotherapy, difficulty with radical resection, limited economic conditions, or personal beliefs, this type of limb salvage is not recommended. By analyzing the data from this study, we could optimize this type of limb salvage in terms of the selection of internal fixation, location of the lesion, surgical technique, and management of postoperative complications.

**Fig. 4:**
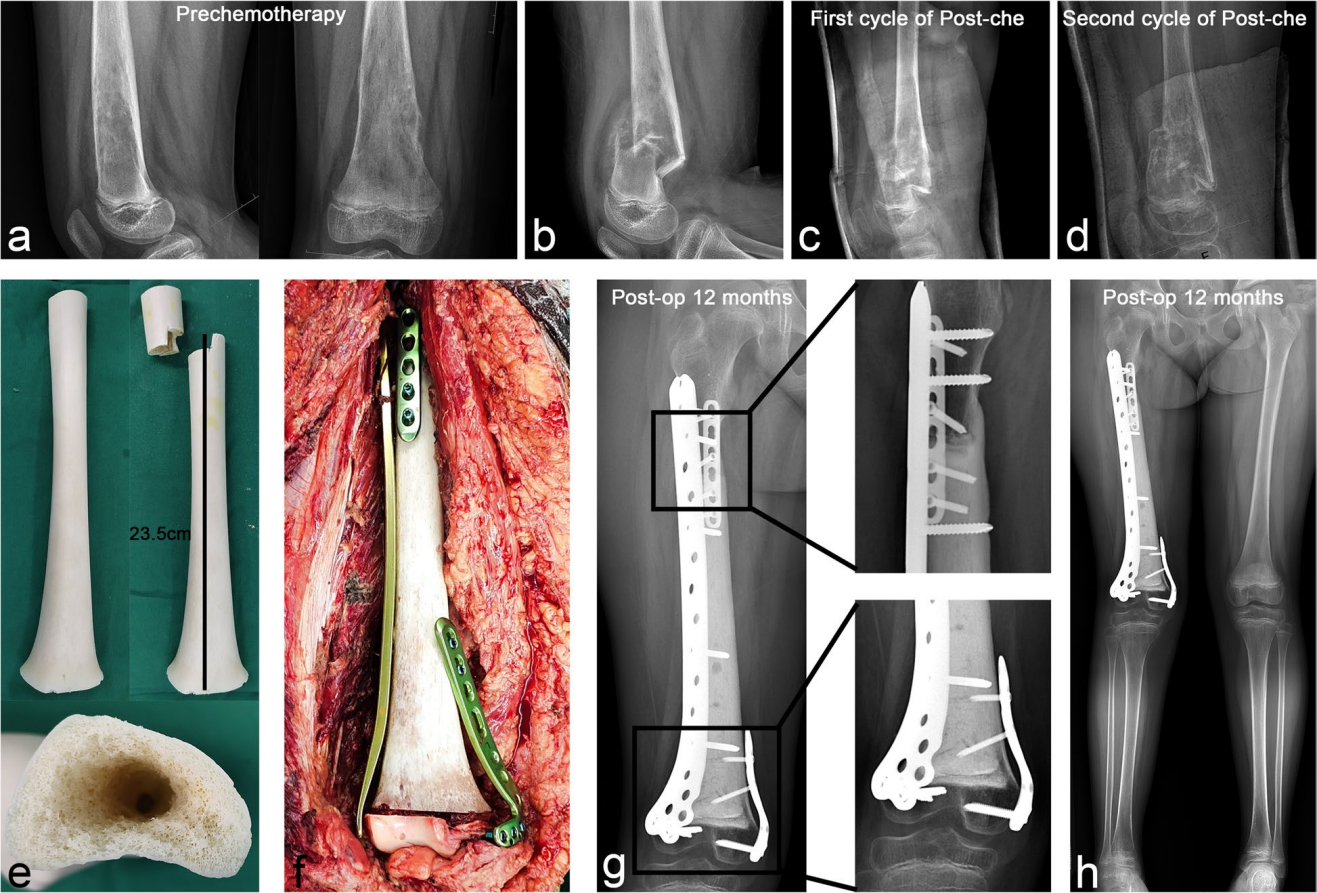
Case 24. A case of stage IIB osteosarcoma in distal femur with pathological fracture. **a**–**d** Anteroposterior and lateral X-rays before and after neoadjuvant chemotherapy showed significant bone repair at the fracture site during chemotherapy. **e** Multidimensional osteotomy was performed at the diaphysis of the allograft to enlarge the host–donor junction surface and empty the medullary cavity for cement filling. **f** The allograft was fixed to the host laterally with a plate spanning the full length of the allograft and was assisted by a short plate anteriorly to the proximal host–donor junction and medially to the distal host–donor junction, respectively. **g** The postoperative X-ray at 12 months showed that the host–donor junction of the diaphysis and metaphysis had healed, and **h** the lower limbs were basically equal in length and had normal force lines

Robust internal fixation is a prerequisite for achieving a stable and gradually activated survival after allograft reconstruction [31–33]; the quality of the host bone is also an important factor affecting bone healing [34]. In the early clinical application of allograft, single long plate or short segment double plate fixation was often used [31, 33], resulting in many mechanical-related complications, such as allograft fracture, internal fixation fracture, and nonunion of osteotomy surface. With the promotion of the application of intramedullary nails, simple intramedullary nailing was once advocated [1, 13, 35]; however, the incidence of bone nonunion could not be effectively reduced. Experience is summarized based on lessons of failure. From previous research [31, 33], we found that robust internal fixation may be the core element for achieving allograft survival, and the stress protection of the internal fixation device to the allograft is often lifelong. Stress protection across the full length of the allograft can effectively avoid the occurrence of postoperative allograft fractures and ensure the stability of the osteotomy surface to avoid nonunion due to the micro-movement of the allograft. In our cohort, we preferred using double long steel plate or intramedullary nail plus steel plate for combined internal fixation (Fig. 4). Especially in the more recent cases of this cohort, we all adhered to the reconstruction concept of robust internal fixation philosophy while choosing the fixation method for allografts.

The location of the focus has a direct impact on the therapeutic effect of allograft in repairing bone defects. A significant difference was found between intercalary allograft reconstruction and osteoarticular allograft reconstruction in the recovery of patients’ limb function. Toy [2] has suggested that osteoarticular allograft reconstruction was most appropriate for restoring bone stock in young patients with expected long-term survival. Muscolo [29] has retrospectively analyzed the data of 52 patients and found that osteoarticular allograft was a valuable reconstruction method for the treatment of proximal tibial large defects after bone tumor resection. Furthermore, its efficacy in repairing bone defects of the distal radius appeared satisfactory [36]. However, osteoarticular allograft reconstruction had some shortcomings, including high rates of mechanical complications, which inevitably required reoperation and prevented the promotion of this reconstruction method [37]. Intercalary reconstruction appears to be significantly superior to osteoarticular reconstruction as regards functional recovery and incidence of complications. Luis [38] has reported that preserving the epiphysis or its joint could avoid joint complications in osteoarticular reconstruction, and the functional recovery of patients was more favorable. In our cohort, many complications were noted in osteoarticular allograft reconstruction (Fig. 1). Six of the 10 cases underwent reoperation due to allograft fracture, nonunion, or osteoarthritis. Since osteoarticular allograft reconstruction sacrifices normal joint accessories, the articular cartilage is inactive and can only be fixed unidirectionally. As a result, it is very susceptible to joint degeneration and mechanical obstacles. Therefore, while osteoarticular reconstruction may be suitable for preserving bone stock, this method may not be effective for long-term reconstruction. Allograft-prosthesis composite (APC) may represent a better option for the preservation of bone stock with excellent functional restoration [39].

**Fig. 5:**
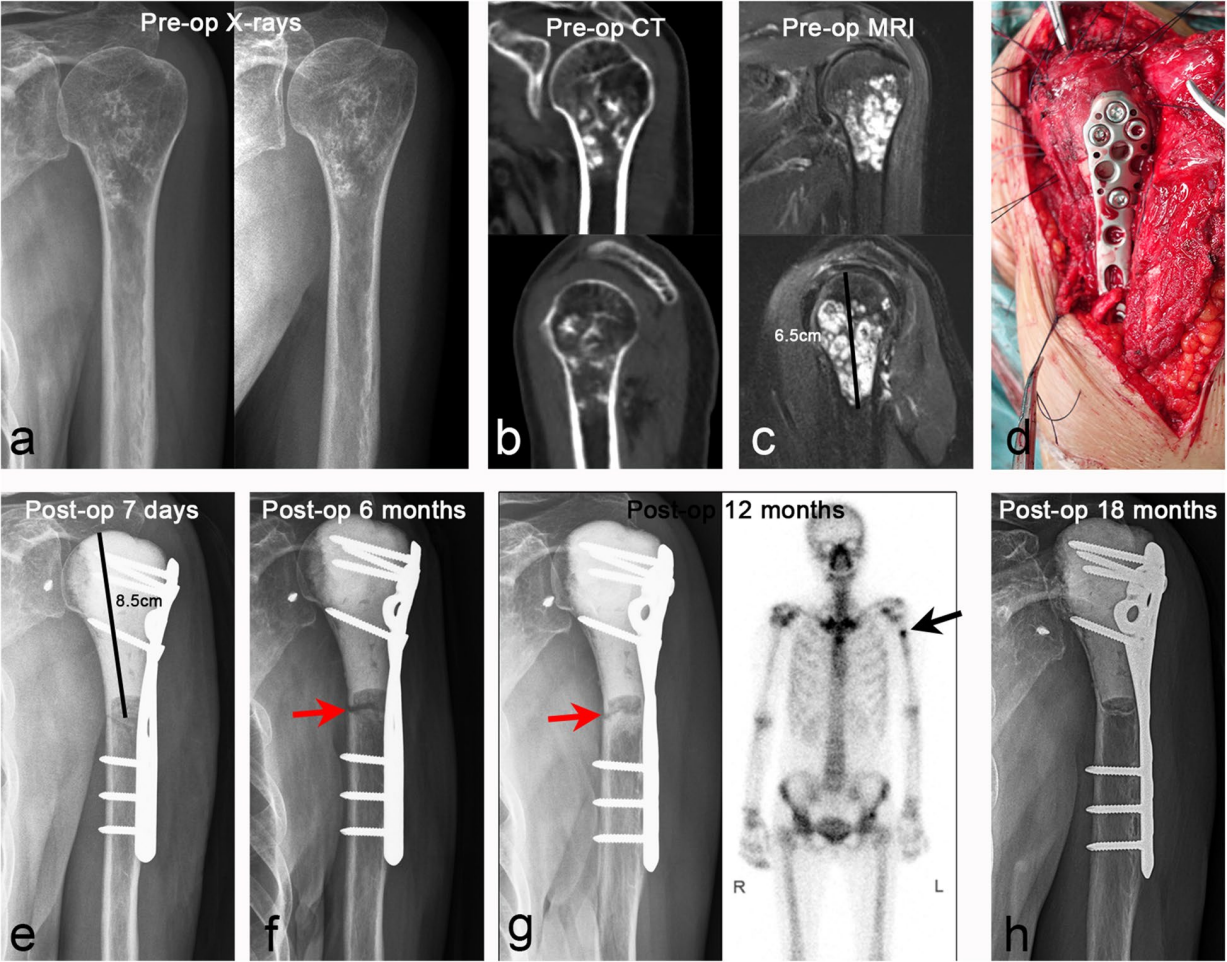
Case 18. A case of stage IIA chondrosarcoma in proximal humerus. **a**–**c** Anteroposterior and lateral X-ray, CT scan, and MRI images. **d** The allograft was fixed with a long segmental plate to repair the bone defect after tumor resection, and the rotator cuff and joint capsule were repaired. **e**–**g** The postoperative anteroposteric X-ray at 7 days, 6 months, and 12 months showed bone resorption of the host–donor junctions at 6 months after operation, and SPECT scan at 12 months showed active metabolism at the host–donor junctions, indicating that the process of bone healing was in progress. **h** The postoperative X-ray at 18 months showed that the host–donor junction was completely healed, and the internal fixation was firmly fixed

**Fig. 6:**
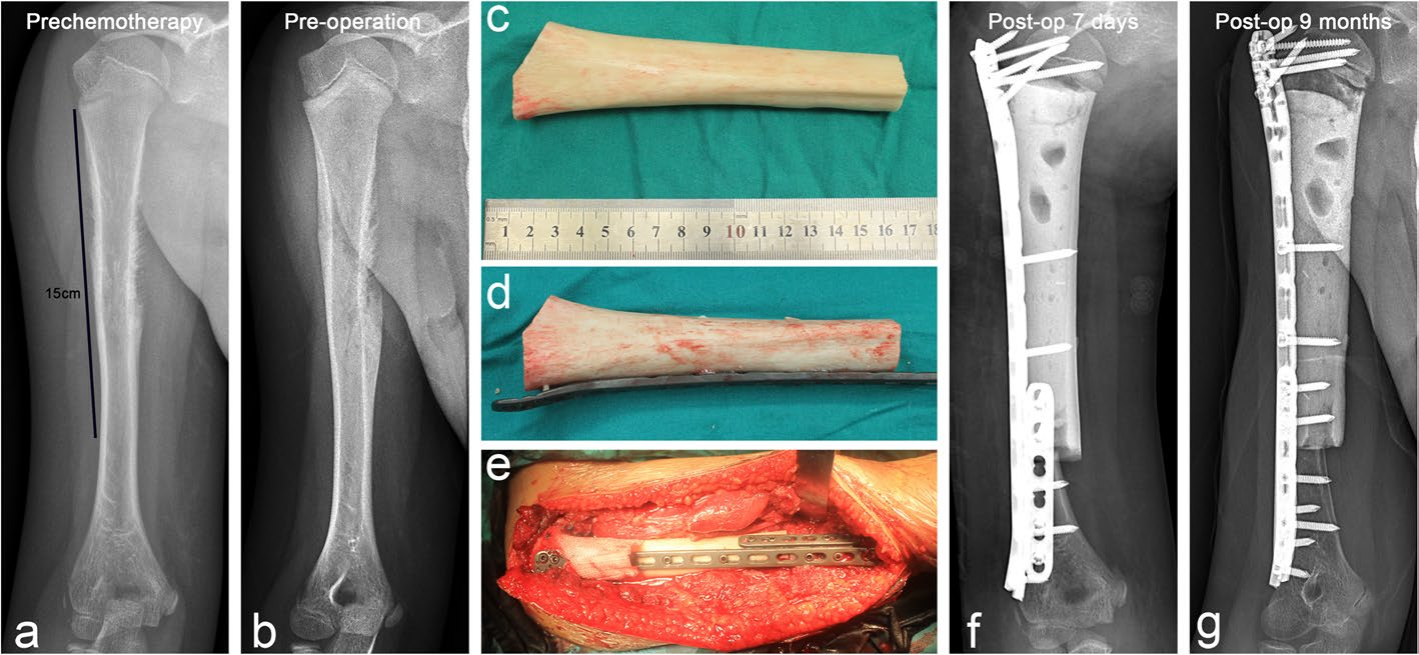
Case 28. A case of stage IIB Ewing’s sarcoma in humeral shaft. **a** and **b** Anteroposterior X-rays before and after neoadjuvant chemotherapy. **c** Allograft for repairing bone defect was filled with bone cement and **d** fixed with steel plate in advance. **e** The allograft was combined with the remaining humeral head and distal humerus, and the distal host–donor junction was reinforced with a short plate. **f** and **g** Anteroposterial X-rays were performed at 7 days and 9 months postoperatively, respectively

**Fig. 7:**
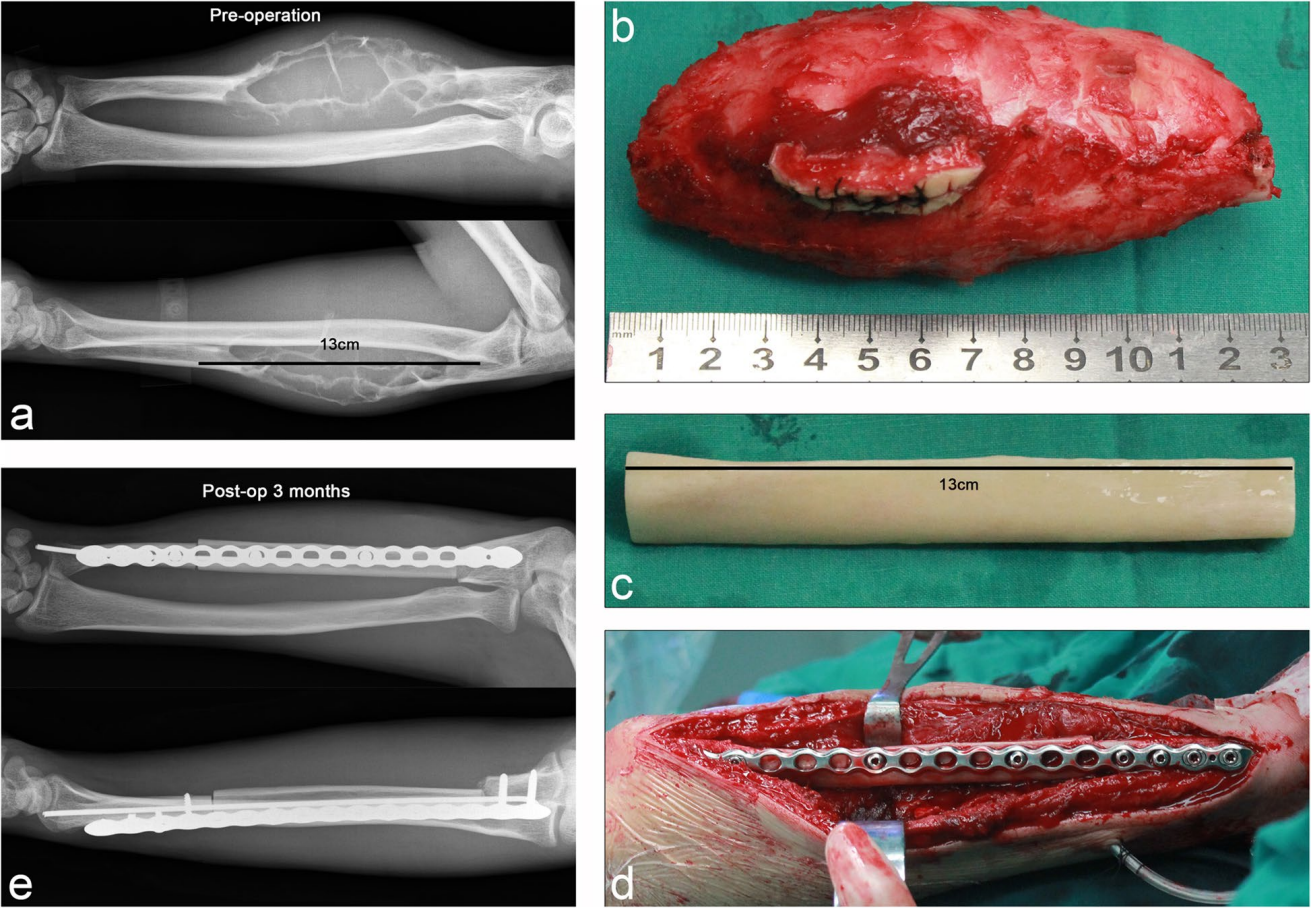
Case 20. A case of stage IIB fibrosarcoma in trunk of ulna. **a** Preoperative anteroposterior and lateral X-ray showed that the tumor was located in the diaphysis with obvious expansion. **b** Gross specimens of resected tumors and **c** the allograft for repairing bone defects. **d** Elastic intramedullary nails and long segmental reconstruction plates were used to secure the allograft to the host. **e** Anteroposterior and lateral X-rays at 3 months postoperatively suggested that the allograft was firmly fixed

Host–donor junction healing, which has always received the most attention, is fundamental to the success of biological reconstruction (Fig. 5). Allografts cannot be completely transformed into host bone, which relies on callus formation and osteogenesis induced by the host to gradually complete the process of crawling replacement [40]. Many studies have reported risk factors for host–donor junction healing [40, 41]. A multicenter study by Bus et al. [35] reported a 40% incidence of nonunion after allograft reconstruction, and the risk of nonunion could be increased by simply using intramedullary nail fixation or allograft length > 10 cm. Lee et al. [42] recommended the use of V-shaped osteotomy to increase the surface of impact between the allograft and the host to accelerate bone healing. The convex-concave connection could create a large surface area at the allograft-host bone connection site while also preventing displacement of the contact surface and enhancing local stability. Furthermore, proper stress stimulation of the host–donor junction is also helpful for bone healing [32, 43, 44]. In our study, the nonunion rate in the diaphysis (18.4%) was higher than in the metaphysis (3.1%). We found that excellent bone healing was determined by many factors, including robust internal fixation, increased contact area of the host–donor junction, optimized matching (Fig. 6), and appropriate stress stimulation to avoid bone absorption (Fig. 7).

Allograft infection is the most fatal complication and major cause of allograft failure. This complication is often difficult to cure without thorough debridement, and severe cases require amputation. The causes of infection are often multifactorial. Gebhardt et al. [45] reported that patients with osteosarcoma, who received adjuvant and neoadjuvant chemotherapy, had an infection rate of 30%. As chemotherapy reduces the patient’s immunity, stricter patient care is required to prevent infection. Furthermore, adequate soft tissue coverage of allografts can effectively reduce the risk of infection [46, 47]. Proper coverage of the allograft can be achieved through the transfer of pedicled muscle flaps. Therefore, in patients with significant soft tissue defects after resection of soft tissue sarcoma involving the bone [48, 49], biological reconstruction can still be used to repair bone defects. In addition, sufficient postoperative wound drainage can effectively reduce the risk of infection and provide a favorable environment for bone induction. Compared to mechanical reconstruction, the allograft biological reconstruction method has good long-term anti-infection ability, which is a unique advantage (Fig. 6).

In this study, we found that there were some deficiencies in the repair of bone defects by allografts, but we also summed up many experiences and lessons. First, the allograft was more suitable for repairing intercalary bone defects, whereas APC may be more appropriate for osteoarticular defects. Second, robust internal fixation and intramedullary cement reinforcement across the full length of the allograft could effectively prevent allograft fracture and nonunion. Third, multidimensional osteotomy can increase the contact surface and stability of the host–donor junction to accelerate bone healing. Fourth, pedicled muscle flap transplantation to cover the allograft and adequate postoperative wound drainage could effectively prevent the occurrence of infection-related complications.

This study has some limitations. First, the sample size was limited, and the occurrence and probability of complications could not be evaluated. Second, this study had a retrospective design and lacked effective case–control analysis. Therefore, we could not effectively compare the prognoses of intercalary and osteoarticular reconstruction. Finally, the follow-up period of this study needs to be further extended to evaluate the long-term efficacy and complications of biological reconstruction.

## Conclusions

Allografts are an excellent choice for repairing bone defects after malignant bone tumor resection and are more suitable for intercalary bone defects of limb extremities. Multidimensional osteotomy, robust internal fixation across the full length of the allograft, intramedullary bone cement reinforcement, and pedicled muscle flap transfer for allograft coverage can effectively improve allograft survival and healing. Furthermore, optimization of treatment details could help effectively reduce postoperative complications and achieve a satisfactory prognosis.

## Supplementary Information

**Additional file 1:** **Supplemental Table.** Demographic and surgical data of patients.

**Additional file 2:** **Supplemental figure.** Kaplan–Meier survival curve for allografts.

**Additional file 3:** **Supplementary videos.** Video 1 Case 21. Video 2 Case 1. Video 3 Case 23. Video 4 Case 20. Video 5 Case 5.

## Data Availability

All the data used in the article can be obtained from the medical record information system of Xiangya Hospital, Central South University. Any questions or enquiries regarding the present study can be directed to Wei Luo, MD, PhD (luowei0928@126.com), as corresponding author.

## Abbreviations

CT: Computed tomography
MRI: Magnetic resonance imaging
MSTS: Musculoskeletal Tumor Society
APC: Allograft-prosthesis composite
IMN: Intramedullary nail
SSP: Single steel plate
DP: Double plate
INCP: Intramedullary nail combined plate
UDPS: Undifferentiated pleomorphic sarcoma
