# Supplementary material for: Biological reconstruction of bone defect after resection of malignant bone tumor by allograft: a single-center retrospective cohort study
Source: World J Surg Oncol. 2023 Jul 31;21:234. doi: 10.1186/s12957-023-03121-7 (PMC10388483; doi:10.1186/s12957-023-03121-7)
Supplement: Supplementary file 1 — Additional file 1: Supplemental Table. Demographic and surgical data of patients. [file 12957_2023_3121_MOESM1_ESM.docx]

**Supplemental Table: Demographic and surgical data of patients**

| **Case** | **Gender** | **Diagnosis** | **Age** | **Enneking stage** | **Reconstruction technique** | **Allograft length(cm)** | **Location** | **Duration of Follow-up (month)** | **Interface matching** | **Complication** | **Method of fixation** | **Preoperative MSTS score** | **Postoperative MSTS score** |
| --- | --- | --- | --- | --- | --- | --- | --- | --- | --- | --- | --- | --- | --- |
|  |  |  |  |  |  |  |  |  |  |  |  |  |  |
| 1 | M | OS | 18 | II B | Intercalary | 12.5 | Femur | 29 | rough | Relapse, Metastasis | INCP | 21 | 28 |
| 2 | F | OS | 14 | II B | Intercalary | 19.8 | Tibia | 87 | precise | Rejection | DP | 22 | 29 |
| 3 | F | CS | 66 | II A | Intercalary | 24.5 | Femur | 103 | rough | / | INCP | 24 | 29 |
| 4 | F | OS | 23 | II B | Intercalary | 14.3 | Femur | 97 | rough | Rejection | INCP | 22 | 25 |
| 5 | F | OS | 16 | II B | Intercalary | 14.5 | Tibia | 112 | rough | / | INCP | 19 | 24 |
| 6 | M | UDPS | 35 | II B | Intercalary | 12.8 | Tibia | 43 | rough | Relapse, Metastasis | IMN | 19 | 26 |
| 7 | F | ES | 28 | II B | Intercalary | 15.5 | Femur | 54 | rough | Metastasis, Nonunion | IMN | 12 | 27 |
| 8 | F | OS | 17 | II B | Intercalary | 14.8 | Tibia | 78 | precise | / | DP | 20 | 28 |
| 9 | F | AB | 36 | II A | Intercalary | 20.5 | Tibia | 89 | rough | / | DP | 24 | 27 |
| 10 | F | OS | 23 | II B | Intercalary | 17.5 | Tibia | 76 | rough | Rejection | INCP | 18 | 25 |
| 11 | F | ES | 26 | II B | Intercalary | 18.5 | Femur | 95 | precise | / | DP | 21 | 26 |
| 12 | M | OS | 18 | II B | Intercalary | 16 | Tibia | 88 | precise | Rejection | INCP | 19 | 28 |
| 13 | F | OS | 19 | II B | Intercalary | 17.5 | Femur | 65 | rough | / | INCP | 18 | 29 |
| 14 | M | OS | 18 | II B | Intercalary | 16.5 | Femur | 25 | rough | Relapse, Metastasis | DP | 22 | 25 |
| 15 | F | OS | 14 | II B | Intercalary | 19.8 | Tibia | 48 | rough | Rejection | INCP | 18 | 29 |
| 16 | F | OS | 25 | II B | Intercalary | 18.8 | Tibia | 32 | rough | Metastasis, Rejection | INCP | 10 | 24 |
| 17 | M | OS | 28 | II B | Intercalary | 15.5 | Femur | 73 | precise | / | INCP | 21 | 29 |
| 18 | M | CS | 53 | II A | Osteoarticular | 12.5 | Humerus | 56 | precise | / | SSP | 19 | 26 |
| 19 | F | OS | 10 | II B | Intercalary | 18.5 | Femur | 47 | precise | Rejection | DP | 17 | 29 |
| 20 | M | FS | 23 | II B | Intercalary | 13 | Ulna | 69 | precise | / | INCP | 22 | 29 |
| 21 | M | OS | 22 | II A | Intercalary | 15.6 | Femur | 64 | rough | Rejection, Nonunion | INCP | 21 | 29 |
| 22 | F | OS | 7 | II B | Intercalary | 14.5 | Femur | 35 | precise | / | DP | 19 | 28 |
| 23 | M | FS | 21 | II A | Osteoarticular | 10.5 | Radius | 48 | rough | Nonunion, Osteoarthritis | SSP | 24 | 26 |
| 24 | F | OS | 10 | II B | Intercalary | 21.5 | Femur | 53 | precise | Metastasis | DP | 8 | 28 |
| 25 | F | ES | 10 | II B | Intercalary | 18.5 | Femur | 26 | precise | / | DP | 15 | 29 |
| 26 | M | OS | 11 | II B | Intercalary | 23 | Femur | 34 | precise | / | DP | 21 | 29 |
| 27 | F | FS | 40 | II A | Osteoarticular | 12.5 | Femur | 32 | rough | Osteoarthritis | SSP | 17 | 25 |
| 28 | F | ES | 8 | II B | Intercalary | 11 | Humerus | 33 | precise | / | DP | 21 | 26 |
| 29 | F | FS | 53 | II B | Intercalary | 15.5 | Femur | 45 | precise | Nonunion, Metastasis | INCP | 18 | 30 |
| 30 | M | OS | 12 | II B | Osteoarticular | 17.5 | Tibia | 47 | rough | Nonunion, Metastasis, Fracture | DP | 18 | 17 |
| 31 | M | ES | 14 | II B | Intercalary | 16.5 | Femur | 49 | precise | / | DP | 21 | 29 |
| 32 | F | OS | 18 | II B | Osteoarticular | 14 | Tibia | 67 | rough | Nonunion, Fracture | DP | 17 | 19 |
| 33 | F | AB | 43 | II A | Intercalary | 16.5 | Femur | 38 | rough | Rejection, Nonunion | DP | 18 | 28 |
| 34 | M | OS | 20 | II B | Intercalary | 17 | Tibia | 74 | precise | / | INCP | 21 | 27 |
| 35 | F | ES | 11 | II B | Intercalary | 14 | Femur | 28 | precise | / | DP | 20 | 29 |
| 36 | M | OS | 15 | II B | Intercalary | 15.5 | Tibia | 32 | precise | / | INCP | 18 | 29 |
| 37 | M | OS | 17 | II B | Intercalary | 20.5 | Femur | 46 | precise | Relapse, Metastasis | DP | 21 | 28 |
| 38 | M | CS | 35 | II B | Osteoarticular | 18 | Humerus | 48 | precise | Nonunion, Fracture | SSP | 23 | 21 |
| ***OS, osteosarcoma; CS, chondrosarcoma; ES, Ewing's sarcoma; UDPS, undifferentiated pleomorphic sarcoma; AB, ameloblastoma; FS,Fibrosarcoma; IMN, intramedullary nail; SSP, single steel plate; DP, double plate; INCP, intramedullary nail combined plate; MSTS, Musculoskeletal Tumour Society scoring system.*** | | | | | | | | | | | | | |
