# Supplementary figures and images for: Biological reconstruction of bone defect after resection of malignant bone tumor by allograft: a single-center retrospective cohort study
Source: World J Surg Oncol. 2023 Jul 31;21:234. doi: 10.1186/s12957-023-03121-7 (PMC10388483; doi:10.1186/s12957-023-03121-7)

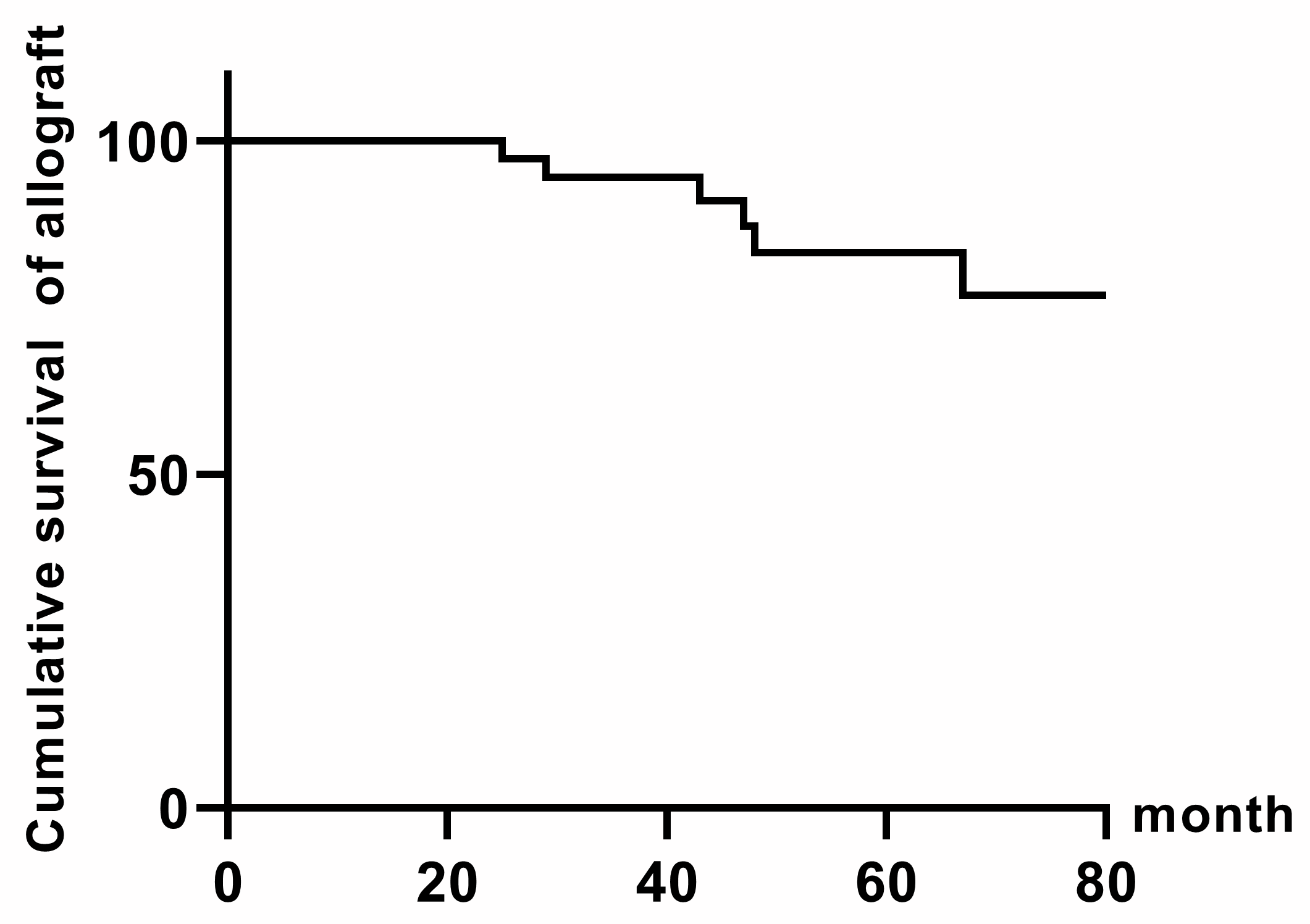


Supplemental figure: Kaplan–Meier survival curve for allografts

Supplement: Supplementary file 2 — Additional file 2: Supplemental figure. Kaplan–Meier survival curve for allografts. [file 12957_2023_3121_MOESM2_ESM.docx]
